# Antennal transcriptome analysis of the chemosensory gene families in the tree killing bark beetles, *Ips typographus* and *Dendroctonus ponderosae* (Coleoptera: Curculionidae: Scolytinae)

**DOI:** 10.1186/1471-2164-14-198

**Published:** 2013-03-21

**Authors:** Martin N Andersson, Ewald Grosse-Wilde, Christopher I Keeling, Jonas M Bengtsson, Macaire MS Yuen, Maria Li, Ylva Hillbur, Jörg Bohlmann, Bill S Hansson, Fredrik Schlyter

**Affiliations:** 1 Chemical Ecology, Department of Plant Protection Biology, Swedish University of Agricultural Sciences, SE-230 53, Alnarp, Sweden; 2 Department of Evolutionary Neuroethology, Max Planck Institute for Chemical Ecology, DE-07745, Jena, Germany; 3 Michael Smith Laboratories, University of British Columbia, Vancouver, BC V6T 1Z4, Canada; 4 Present address: Department of Biology, Lund University, SE-223 62, Lund, Sweden; 5 Present address: Research and Innovation Centre, Fondazione Edmund Mach, San Michele all’Adige, Italy

**Keywords:** *Ips typographus*, *Dendroctonus ponderosae*, Gene ontology, Transcriptome, Odorant receptor, Ionotropic receptor, Gustatory receptor, Odorant binding protein, Chemosensory Protein, Sensory neuron membrane protein

## Abstract

**Background:**

The European spruce bark beetle, *Ips typographus*, and the North American mountain pine beetle, *Dendroctonus ponderosae* (Coleoptera: Curculionidae: Scolytinae), are severe pests of coniferous forests. Both bark beetle species utilize aggregation pheromones to coordinate mass-attacks on host trees, while odorants from host and non-host trees modulate the pheromone response. Thus, the bark beetle olfactory sense is of utmost importance for fitness. However, information on the genes underlying olfactory detection has been lacking in bark beetles and is limited in Coleoptera. We assembled antennal transcriptomes from next-generation sequencing of *I. typographus* and *D. ponderosae* to identify members of the major chemosensory multi-gene families.

**Results:**

Gene ontology (GO) annotation indicated that the relative abundance of transcripts associated with specific GO terms was highly similar in the two species. Transcripts with terms related to olfactory function were found in both species. Focusing on the chemosensory gene families, we identified 15 putative odorant binding proteins (OBP), 6 chemosensory proteins (CSP), 3 sensory neuron membrane proteins (SNMP), 43 odorant receptors (OR), 6 gustatory receptors (GR), and 7 ionotropic receptors (IR) in *I. typographus*; and 31 putative OBPs, 11 CSPs, 3 SNMPs, 49 ORs, 2 GRs, and 15 IRs in *D. ponderosae*. Predicted protein sequences were compared with counterparts in the flour beetle, *Tribolium castaneum,* the cerambycid beetle, *Megacyllene caryae*, and the fruit fly, *Drosophila melanogaster*. The most notable result was found among the ORs, for which large bark beetle-specific expansions were found. However, some clades contained receptors from all four beetle species, indicating a degree of conservation among some coleopteran OR lineages*.* Putative GRs for carbon dioxide and orthologues for the conserved antennal IRs were included in the identified receptor sets.

**Conclusions:**

The protein families important for chemoreception have now been identified in three coleopteran species (four species for the ORs). Thus, this study allows for improved evolutionary analyses of coleopteran olfaction. Identification of these proteins in two of the most destructive forest pests, sharing many semiochemicals, is especially important as they might represent novel targets for population control.

## Background

The European spruce bark beetle, *Ips typographus* L., and the North American mountain pine beetle, *Dendroctonus ponderosae* Hopkins (Coleoptera: Curculionidae: Scolytinae) are serious pests of coniferous forests. *I. typographus* mainly attacks Norway spruce (*Picea abies*) in Eurasia, whereas *D. ponderosae* infests several species of pine in western North America. Currently, large-scale *D. ponderosae* outbreaks have resulted in unprecedented economic losses and turned North American forests into major sources of carbon release [1]. The olfactory sense drives bark beetle behaviors that are important for fitness, such as the localization of suitable hosts and mates [2]. In the search for suitable host material, bark beetles respond to volatiles that emanate from both host and non-host plants [3-5]. However, most individuals locate trees by means of an aggregation pheromone that is released by beetles that have already attacked the tree. This signal is responsible for coordinated mass-attacks, which often lead to the death of the host tree [2] and large-scale forest destruction. Due to their ecological and economic impact, an extensive knowledge base on bark beetle chemical ecology and olfactory physiology has been established [2,3,6,7]. However, information on the molecular aspects of odor detection has been lacking until now.

In insects, volatile molecules are detected by olfactory sensory neurons (OSNs) that are housed within special structures (sensilla) predominantly on the antennae, and to a lesser extent on the maxillary palps. The cell membrane of OSNs contains receptor proteins that bind odor ligands [8,9]. The binding of a ligand to a receptor protein is the key event in olfactory transduction, as it converts a chemical signal in the environment into an electrical signal that can be interpreted by the insect nervous system [10].

Receptors from three large and divergent multigene families are expressed in insect OSNs [9,11-13], namely the odorant receptors (OR), ionotropic receptors (IR), and gustatory receptors (GR), the latter group notably containing carbon dioxide-detecting receptors [14,15]. However, most GRs are expressed in gustatory receptor neurons in taste organs and are involved in contact chemoreception. These GRs typically detect different sugars, bitter compounds, and contact pheromones [12].

Insect ORs are seven-transmembrane domain proteins [16,17] with a reversed membrane topology (intracellular N-terminus) compared to vertebrate ORs, which are G-protein coupled receptors [18]. Insect ORs and GRs are distantly related members of the same superfamily [19]. In general, ORs (and GRs) show little sequence homology to each other and they are unrelated to vertebrate ORs. The conventional exchangeable OR that determines ligand specificity [20] forms heteromers of unknown stoichiometry with a conserved co-receptor, known as Orco [21]. Orco is ubiquitously expressed in OSNs that express ORs [22,23] and necessary for olfactory responses and for localization of the conventional OR in the cell membrane [18,24]. Putative insect ORs have been identified mainly in species with sequenced genomes [8,25]. Recently, however, studies on antennal transcriptomes have led to the identification of OR sets in several moth species [26-28] and one beetle [29]. The ORs respond to a variety of volatile chemicals [20,30], including pheromones [31] and plant or microbe-derived compounds [32]. Some ORs are highly defined in their response specificity [32], whereas others appear more broadly tuned, especially at high stimulus concentrations [20].

IRs were recently discovered as another class of receptors involved in chemoreception [9]. They are related to ionotropic glutamate receptors (iGluRs) that function in synapse communication, but have atypical binding domains. IRs have been identified throughout protostome lineages (including arthropods, mollusks, annelids and nematodes) and, thus, constitute a far more ancient group of receptors than the ORs [33]. IRs form complexes with up to three subunits, including odor-specific receptors and one or two broadly expressed co-receptors [34]. In insects, the IRs are divided into two major groups: the “antennal IRs” that have an olfactory function and are conserved across insect orders, and the species-specific “divergent IRs”, some of which have been assigned a tentative role in taste [33]. Antennal IRs in *Drosophila* have different odor specificity compared to the ORs and respond to nitrogen-containing compounds (e.g. ammonia and amines), acids, and aromatics (i.e. phenylacetaldehyde) [34].

In addition to the receptor genes, other multigene families encode proteins with critical roles in olfaction. Odorant binding proteins (OBP) are small soluble proteins (generally 135–220 amino acids long) with two or three disulfide bridges [35,36]. OBPs are highly abundant in the sensillar lymph of insects and are thought to solubilize hydrophobic molecules and deliver them to the receptors [35]. Studies have shown conflicting results whether or not OBPs affect the response specificity of OSNs [37]. At least in some studies, the specificity of pheromone receptors was improved by the presence of OBPs (i.e. a class of OBPs called pheromone binding proteins, PBPs) [38,39]. Some evidence suggests that OBPs might undergo odor-induced conformational changes, with a change in the OBP itself triggering the response of the OSN [40]. In insects with sequenced genomes, the number of OBP coding genes normally ranges from ca. 40–60 [35].

Chemosensory proteins (CSP) constitute another class of small binding proteins (ca. 130 amino acids long). They are more conserved than OBPs and are characterized by the presence of 4 cysteines that form two disulfide bridges [41]. CSPs may have shared a common ancestor with the OBPs near the origin of the arthropods [42]. Like OBPs, CSPs are present in high concentration in chemosensory sensilla (outer sensillum lymph [43,44]). However, the majority of them are also expressed in various non-sensory tissues [45] and they seem to play a role in development, moulting [45], and leg regeneration [46]. Some CSPs bind pheromone compounds [41,47], but their exact role in chemosensory systems remains uncertain [35,48]. Most *Drosophila* genomes contain only 4 CSP coding genes and *T. castaneum* has 20 [35]. The genome of *Aedes aegypti* mosquitoes contains 43 members of this family, the largest number found in insects so far [49]*.*

Finally, the sensory neuron membrane proteins (SNMP) are proteins of the CD36 family that associate with pheromone-responding OSNs [50]. Their functional significance is still poorly understood, but SNMP is crucial for proper pheromone detection in *D. melanogaster* and also required for activation of a pheromone receptor in *Heliothis virescens* moths [51]. In contrast, SNMP was dispensable for responses of a fly receptor (DmelOR22a) to fruit-related esters [51]. Insects generally have two representatives of SNMPs (SNMP1 and SNMP2), although copy numbers of each orthologue seem to vary somewhat across species [52].

In the largest insect order, Coleoptera, ORs have been identified from only two species: from the genome of the red flour beetle, *Tribolium castaneum*[53], and recently from the antennal transcriptome of the cerambycid beetle, *Megacyllene caryae*[29]. Members of the other olfactory gene families have been identified only in *T. castaneum*[33,35,52-54]. Consequently, additional beetle species need to be investigated to reach a better understanding on the molecular biology of coleopteran and insect olfaction.

In this study, we assembled and analyzed bark beetle antennal transcriptomes from next-generation sequencing. We report the results from gene ontology (GO) annotation as well as sets of putative OBPs*,* CSPs*,* SNMPs*,* ORs, GRs*,* and IRs in *I. typographus* and *D. ponderosae*. Identification of the chemosensory genes in these devastating insect pests is especially relevant because of their potential as novel targets for pest control.

## Methods

### Mountain pine beetle

The source of *D. ponderosae* (obtained from lodgepole pine, *Pinus contorta*) antennal tissue and the method of sequencing have been reported previously in a larger transcriptome study [55]. From this transcriptome dataset originating from several tissue types, sequences originating only from a non-normalized antenna-specific cDNA library were re-assembled for the analyses presented in the present study. This included 12,142 paired-end Sanger reads, 1,147 single-end Sanger reads and 1,048,708 Roche 454 reads. Newbler (version 2.6, Roche) was used for assembly using the "-cdna" switch for transcriptome assemblies and a 45 bp cutoff to eliminate short reads. For identification of OBPs, CSPs, and SNMPs in *D. ponderosae* we also used a combination of Sanger-specific (CAP3 assembly) data and transcriptome assemblies from other tissues and life stages, since these proteins may have sensory or non-sensory functions in non-antennal tissues. We did not have such assemblies for *I. typographus.*

### European spruce bark beetle

#### Insects, RNA extraction and cDNA synthesis

*I. typographus* was reared on Norway spruce (*Picea abies*) logs in an environmental chamber (25°C, 70% RH, 20:4 L:D photoperiod) [56], starting from individuals collected from their natural habitat near Asa and Älmhult, southern Sweden. Emerged adults were kept in a state of low activity in a refrigerator (1–5°C) before being used for RNA extraction.

Two hundred adult *I. typographus* were collected in a 50 ml plastic tube, approximately two weeks after their emergence. The tube was submerged in liquid nitrogen, after which it was vigorously shaken using a vortex shaker to separate extremities from the body. Body parts were suspended in -20°C acetone (>99%, Fisher Scientific AB, Sweden) and passed through meshes that filtered out the antennae. After removal of the acetone, 0.6 ml TRI reagent (Ambion) was added to the antennae and the sample was homogenized using a Tissue-tearor (model 98370–365, Bartlesville, OK, USA). Total RNA was extracted following the TRIZOL protocol, but using 1-bromo-3-chloropropane instead of chloroform. 1.7 μg total RNA was sent to Evrogen (Moscow, Russia) for synthesis of duplex-specific-nuclease normalized cDNA [57,58].

#### Sequencing and assembly

The *I. typographus* cDNA was sequenced at LGC Genomics (Berlin, Germany), using 454 GS/FLX sequencing (Roche Applied Science, Mannheim, Germany) with titanium chemistry, to produce 350,000 reads for a total of 114 megabases. Furthermore, Illumina sequencing was performed at the Max Planck Institute for Molecular Genetics in Berlin to generate a further 3.6 million reads for a total of 122 megabases.

Short or low-quality reads, as well as linker and adapter sequences were removed by the Crossmatch program (454 reads, Incogen Inc., Williamsburg, VA, USA) or by the built-in sequence cleanup of Seqman Ngen (Illumina reads, DNAStar, Madison, WI, USA). The 454 reads were assembled using Seqman Ngen to generate a backbone; subsequently, the Illumina reads were mapped onto this backbone using Seqman Ngen to correct for technology-inherent read errors. The resultant contigs were annotated using a Codequest Workstation (Timelogic/Active Motif, Carlsbad, CA) [27].

### Annotation

For an initial assessment of the two assembled beetle antennal transcriptomes, gene ontology (GO) annotation was performed using Blast2GO [59,60]. Blast2GO annotation associates genes or transcripts with GO terms using hierarchical vocabularies. Genes are described in terms related to molecular function, biological process, or cellular component, allowing for meta-analyses of gene populations [27,61].

The BLAST step was performed with a lenient E-value cutoff at 0.1 to account for the high sequence variability among the olfactory gene families. The mapping step was done using default settings, whereas a lenient E-value (0.1) and lower annotation cut-off (55) and GO-weight (5) were used in the first annotation step to increase the proportion of annotated transcripts. Annotation was further enhanced by merging annotation with results of InterProScan database search at the EBI [62], ANNEX procedure, and the Blast2GO validation step. A subsequent GO-slim step was not used, as this procedure removed the low frequency odorant protein families from the annotation.

For annotation of ORs, IRs, GRs, OBPs, CSPs, and SNMPs in *I. typographus* and *D*. *ponderosae*, contigs were analyzed with tBLASTx searches against custom-made databases and the non-redundant nucleotide collection at NCBI. Additionally, HMM-based searches of the PANTHER database of domain family profiles were done. We identified non-redundant translated proteins with reciprocal BLAST using the comprehensive datasets available for OBPs and CSPs [42], as well as SNMPs [52].

For contigs/isotigs with hits against genes of interest, open reading frames were identified and the annotation verified by additional BLAST (http://blast.ncbi.nlm.nih.gov/Blast.cgi) searches. Contigs containing suspected sequencing errors (mainly insertions/deletions in homopolymer regions) were edited manually after identifying miss-assemblies through manual inspection of the assembly files, ESTs, or genomic data (*D. ponderosae*) [63]. The suffix “FIX” was added to the gene name of such edited sequences, and also to those extended by RACE-PCR (below).

TMHMM 2.0 (http://www.cbs.dtu.dk/services/TMHMM/) was used to predict transmembrane domains of candidate ORs, IRs, and GRs. For all proteins studied, amino acid sequences were aligned using MAFFT [64], and maximum-likelihood analysis and dendrogram construction were subsequently performed with FastTree [65]. Dendrograms were colored and arranged in FigTree (http://tree.bio.ed.ac.uk/software/figtree/). To ensure that sequences corresponded to unigenes (and not to fragments of the same gene), only those that showed sufficient overlap in multiple sequence alignments were included in the analysis. In addition, for contigs that shared >98.5% amino acid identity only one “copy” (the contig with the longest ORF) was included. *I. typographus* 454- and Illumina sequences have been submitted to EBI (project accession number ERP001792). The *D. ponderosae* antennal Sanger and 454 sequence data have previously been submitted to NCBI (accession numbers GT344964-GT358252 and SRX132062, respectively). All bark beetle contigs/isotigs have been submitted to the Transcriptome Shotgun Assembly (TSA) sequence database at NCBI (accession numbers GACR00000000 and GABX00000000 for *I. typographus* and *D. ponderosae*, respectively) or to GenBank (*D. ponderosae* genes with representative full-length cDNA clones) (see Additional file 1 for accession numbers for the individual olfactory genes).

### RACE-PCR

The assembled contigs from the 454- and Illumina sequencing of the *Ips* transcriptome did not always constitute full-length transcripts. Therefore, for better resolution of phylogenetic analyses, some sequences encoding putative ORs were elongated using RACE-PCR (Rapid Amplification of cDNA Ends; SMARTer cDNA amplification kit, Clontech) with a nested protocol following the manufacturer’s instructions. Total RNA from 300 adult beetle antennae (extracted using RNeasy MiniKit, Qiagen) was used as template to generate RACE-ready cDNA. Primer design was performed manually, but aided with Tm-calculations and self-complementarity checks using Oligo Calc (http://www.basic.northwestern.edu/biotools/OligoCalc.html). Amplified and extended DNA was cloned (TOPO TA cloning kit dual promoter, PCRII®-TOPO® vector, Invitrogen) before being sequenced (Eurofins MWG Operon, Ebersberg, Germany).

## Results

### Assembly

The *D. ponderosae* antenna-specific assembly resulted in 19,523 isotigs from 15,736 isogroups and 19,343 singletons, of which 48 were Sanger reads. The isotigs assembled by Newbler were comparable with the contigs generated by other assemblers, with the exception that Newbler also considers alternative splice variants when producing the isotigs, and these are grouped into different isogroups. The N50 was 1,864 bp and the largest isotig was 8,483 bp. The *I. typographus* assembly resulted in 20,298 contigs with an N50 of 717 bp. The largest contig was 3,389 bp.

### Gene ontology annotation

**Figure 1:**
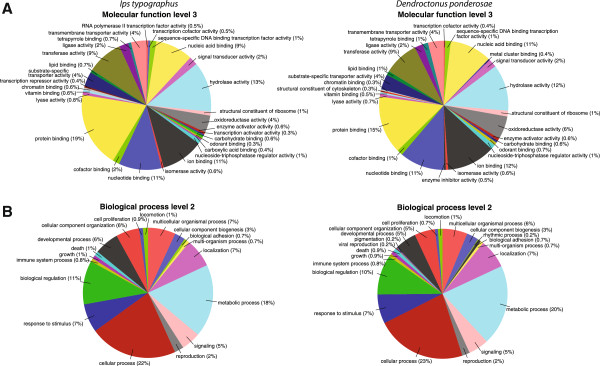
**Gene ontology (GO) results.** GO analysis corresponding to 8,713 contig sequences in *Ips typographus* and 10,713 isotigs in *Dendroctonus ponderosae*, as predicted for their involvement in **A**) molecular function (level 3 GO categorization) and **B**) biological process (level 2). For results presented as detailed bar diagrams, see Additional file 2.

GO annotation indicated that the analyzed antennal transcriptomes of the two bark beetle species were highly similar with respect to GO terms (Figure 1, Additional file 2). In *I. typographus*, 8,713 contigs (43%) were associated with GO terms. In *D. ponderosae*, this number was 10,713 (55%). Thus, a substantial proportion of contigs in both species was not associated with any GO term, and possibly these contigs represent orphan genes. Among the annotated contigs, GO terms related to basic cell functions were the most abundant; however, contigs with GO terms related to olfaction were also present, such as “odorant binding”, “signal transducer activity” (Figure 1A), and “response to stimulus” (Figure 1B). Contigs with GO terms associated with enzymatic activity were well represented, such as “hydrolase activity” and “transferase activity” (Figure 1A).

### Nonreceptor olfactory gene families

We identified 15 transcripts encoding putative OBPs in *I. typographus*, and 31 transcripts in *D. ponderosae*. All but five transcripts (ItypOBP1, 8, 9, 11, and 13) corresponded to full-length genes. One third of the transcripts identified in *D. ponderosae* were not found in the antennal cDNA library, but rather in the cDNA libraries from other body parts (Additional file 3).

**Figure 2:**
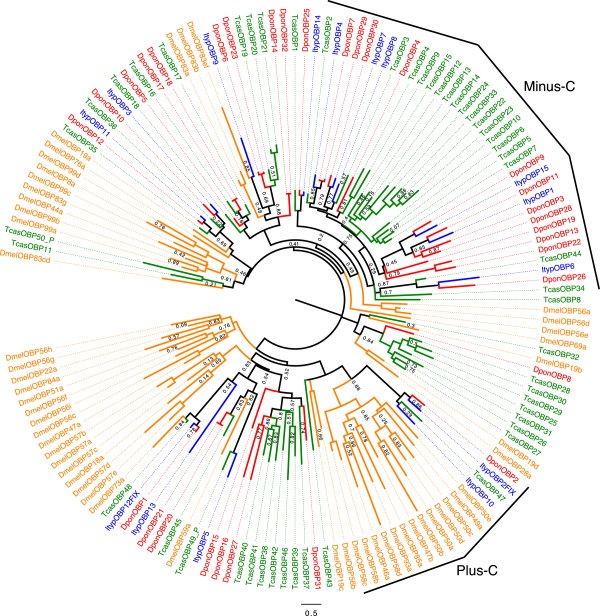
**Maximum-likelihood dendrogram based on protein sequences of candidate odorant binding proteins (OBPs).** Included are OBPs from *Ips typographus* (Ityp), *Dendroctonus ponderosae* (Dpon), *Tribolium castaneum* (Tcas) and *Drosophila melanogaster* (Dmel). One major beetle-specific expansion of Minus-C OBPs is evident. Bark beetle proteins in this expansion have lost cysteine residue C2 and C5. Two OBPs in *I. typographus* and one in *D. ponderosae* belong to the Plus-C group characterized by additional cysteine residues. Numbers refer to support values, which are only displayed when <; 0.9.

In general, OBPs can be classified into different phylogenetic groups. Classic OBPs are characterized by 6 cysteine residues at conserved positions. The Plus-C class has 4–6 additional cysteines and one characteristic proline, whereas the Minus-C class has lost cysteine residues, generally C2 and C5 [35,66]. In our sequence similarity dendrogram, the classic bark beetle OBPs were spread out on various branches (Figure 2) where they generally formed small subgroups together with OBPs mostly from *T. castaneum*.

Two OBPs in *I. typographus* (ItypOBP2 and ItypOBP10) and one OBP in *D. ponderosae* (DponOBP2) were of the Plus-C type and were grouped together with the Plus-C OBP (TcasOBP47) from *T. castaneum* (Figure 2). ItypOBP2 and DponOBP2 shared 45% amino acid identity. Members of the Minus-C class, i.e. 12 DponOBPs, 6 ItypOBPs, and 18 TcasOBPs, formed a large clade (Figure 2). Within this clade, we found a bark beetle-specific expansion, containing ItypOBP1, ItypOBP15, DponOBP3, DponOBP9, DponOBP11, DponOBP13, DponOBP19, DponOBP22, and DponOBP28. All bark beetle full-length Minus-C OBPs had lost C2 and C5.

Six bark beetle OBP orthologous pairs shared >50% amino acid identity between species (ItypOBP14/DponOBP25: 78%; ItypOBP11/DponOBP12: 76%; ItypOBP12FIX/DponOBP1: 74%; ItypOBP7/DponOBP30: 60%; ItypOBP4/DponOBP7: 57%; ItypOBP3/DponOBP10: 51%). There were several OBP pairs with high amino acid identity in *D. ponderosae* (DponOBP14/DponOBP32: 98% identity; DponOBP20/DponOBP21: 97%; DponOBP17/DponOBP18: 96%; DponOBP7/DponOBP29: 91%).

**Figure 3:**
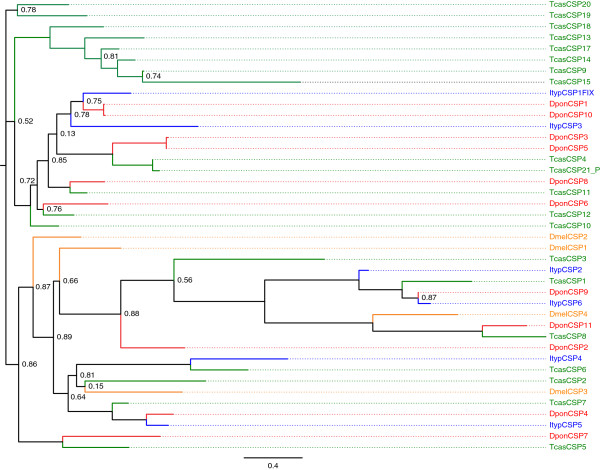
**Maximum-likelihood dendrogram based on protein sequences of candidate chemosensory proteins (CSPs).** Included are CSPs from *Ips typographus* (Ityp), *Dendroctonus ponderosae* (Dpon), *Tribolium castaneum* (Tcas) and *Drosophila melanogaster* (Dmel). Numbers refer to support values, which are only displayed when <; 0.9.

We identified 6 transcripts encoding putative CSPs in *I. typographus*, and 11 transcripts in *D. ponderosae* (Figure 3). Five of the transcripts encoded partial proteins (ItypCSP1-4 and 6, of which 1 and 4 lacked only a few C-terminal amino acids), whereas all the others represented full-length genes. Four of the transcripts identified in *D. ponderosae* were not found in the antennal cDNA library, but rather in the cDNA libraries from other body parts (Additional file 4). The bark beetle CSPs were present on different branches throughout the dendrogram (Figure 3), and no major bark beetle-specific expansion of CSP lineages was evident. Amino acid identity among candidate simple orthologues in the two bark beetles was high (e.g. ItypCSP6/DponCSP9: 91%; ItypCSP5/DponCSP4: 75%; ItypCSP1FIX/DponCSP1: 64%). Two CSP pairs in *D. ponderosae* (DponCSP3/DponCSP5 and DponCSP1/DponCSP10) had the highest (98%) amino acid identity.

**Figure 4:**
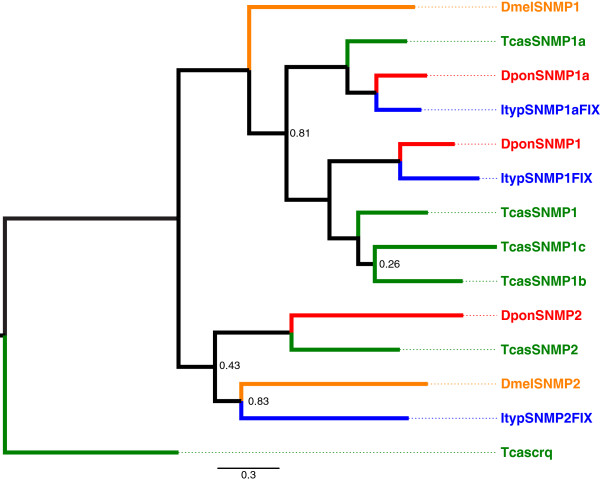
**Maximum-likelihood dendrogram based on protein sequences of candidate sensory neuron membrane proteins (SNMPs).** Included are SNMPs from *Ips typographus* (Ityp), *Dendroctonus ponderosae* (Dpon), *Tribolium castaneum* (Tcas) and *Drosophila melanogaster* (Dmel). The *T. castaneum* orthologue of Croqemort, a non-SNMP member of the CD36 family, was used as outgroup to root the tree. Numbers refer to support values, which are only displayed when <; 0.9.

In each bark beetle species, we found two orthologues of SNMP1 (SNMP1 and SNMP1a)*,* and one orthologue of SNMP2 (Figure 4). ItypSNMP1a was present only as a fragment, whereas transcripts for the others likely represented full-length or very close to full-length genes. The bark beetle SNMPs grouped together with orthologues in *T. castaneum*, with the exception of ItypSNMP2 that paired up with SNMP2 in *D. melanogaster.* SNMP1 and SNMP1a appeared more conserved across the two bark beetles with 58% and 66% amino acid identity, respectively, compared to the SNMP2 orthologues that shared 28% identity.

### Receptor encoding genes

#### Odorant receptors

Similar numbers of putative OR encoding transcripts were annotated in the two bark beetle species. We identified 43 OR candidates in *I. typographus*. Eleven of these were likely representing full-length genes, encoding proteins with more than 374 amino acids. Partial transcripts encoding ItypOR6, 7, 12, 13, 19, 31, 36, and 43 were extended by 3’-RACE-PCR. In *D. ponderosae*, the number of candidate OR transcripts was 49, and the number of full-length candidates was 27. In addition, 4 short partial transcripts in *I. typographus* and 6 in *D. ponderosae* were left unlabeled and excluded from analysis, since unigene identity could not be conclusively confirmed. The shortest partial OR candidate included was ItypOR38 (62 amino acids). Two pairs of receptors, i.e. ItypOR17 and ItypOR24, as well as ItypOR36 and ItypOR39, showed the highest amino acid identity (98% for both pairs).

**Figure 5:**
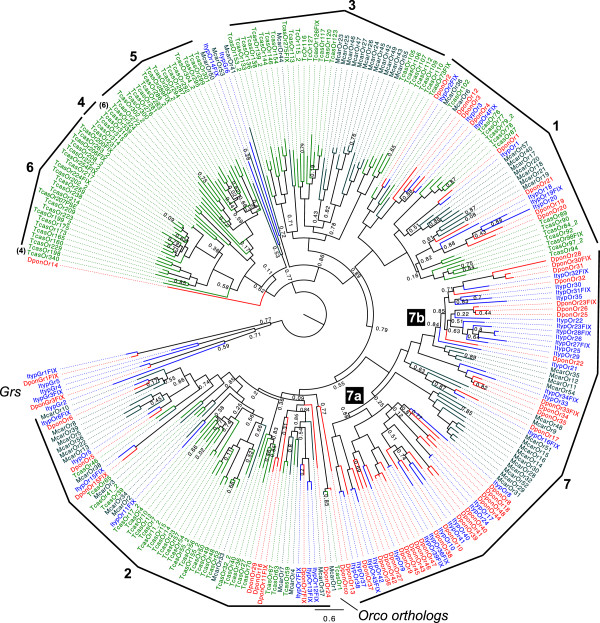
**Maximum-likelihood dendrogram based on protein sequences of candidate odorant receptors (ORs) and gustatory receptors (GRs).** Included are ORs and GRs from *Ips typographus* (Ityp), *Dendroctonus ponderosae* (Dpon), *Tribolium castaneum* (Tcas) and *Megacyllene caryae* (Mcar). The branch containing bark beetle GRs was used as outgroup to root the tree. The different subgroups (numbered 1–7 according to [29,53], and 7a-7b) are discussed in the main text. Originally, TcasOr339 and TcasOr340 were found within group 4 and 6 [53], as indicated here by the numbers in brackets. Numbers at nodes refer to support values, which are only displayed when <; 0.9.

Sequences of the bark beetle ORs were compared with those of *M. caryae* and *T. castaneum*. For the latter species we included only those ORs with confirmed expression in the adult head [53]. Several OR subgroups of various size and content could be distinguished (Figure 5). In order to standardize the numbering of coleopteran OR subfamilies, we numbered these subgroups from 1 to 7 according to previous studies [29,53]. The majority (59 ORs, 64%) of bark beetle ORs were present within group 7, which also contained 16 ORs from *M. caryae*, but no ORs from *T. castaneum*. Fifty-one of these bark beetle ORs formed two subgroups (group 7a and 7b, with 31 and 20 ORs, respectively) that were completely devoid of receptors from the other two beetle species. However, considering only the bark beetle ORs, only minor species-specific subgroups (containing 3–5 ORs) could be seen and they were found within group 7a and 7b (Figure 5). In addition to the bark beetle specific subgroups, group 7 also contained two *M. caryae-*specific subgroups (with 9 and 4 McarORs, respectively) that each formed a sister group to either of the two bark beetle specific subgroups. Finally, a fifth subgroup within group 7 contained ORs from all three species, indicating conservation of some OR sequences among the three xylophagous species. The complete lack of *T. castaneum* receptors within group 7, and the presence of specific subgroups in the other species, indicate broad expansions of OR lineages in bark beetles and cerambycids and/or losses of corresponding OR lineages in *T. castaneum*.

Expansions of OR lineages were also seen in *T. castaneum*. Forty-five (41%) TcasORs formed a large group that was exclusive to the flour beetle. Within this group, the previously defined coleopteran OR subgroups 4–6 could be found [53]. These subgroups were collectively rooted by a smaller clade containing receptors from *I. typographus* and *M. caryae*.

Receptor group 3 contained ORs only from *T. castaneum* and *M. caryae*. The lack of bark beetle ORs in this group suggested that these OR lineages have been lost in bark beetles (or not represented in our transcriptome assemblies), while retained in cerambycids. Within group 3, subgroups that were specific for *T. castaneum* or specific for *M. caryae* could be found*.*

The dendrogram also contained two groups (1 and 2) with OR representatives from all four species (although group 2 was dominated by expanded *T. castaneum* lineages). We found most of the candidate 1:1 orthologous relationships among the bark beetle ORs within groups 1 and 2 (and a few in subgroup 7a) (e.g. Ityp/DponOR1-10, 15). For these candidate orthologous pairs, amino acid identity was 54–69% (excluding fragments <;100 amino acids, i.e. OR4 and OR8). The Orco orthologues rooted group 2.

The co-receptor Orco was identified in the antenna-specific assembly of *D. ponderosae*, but surprisingly not in the antennal transcriptome assembly of *I. typographus*. However, by using PCR with primers designed from a conserved region close to the C-terminus of the DponOrco, we amplified a 62 amino acid fragment of Orco from *I. typographus* antennal-specific cDNA. This ItypOrco fragment shared 97% amino acid identity with DponOrco. As expected, Orco in *D. ponderosae* shared high amino acid identity with Orco orthologues in *M. caryae* (McarOR1*,* 85% identity) and *T. castaneum* (TcasOR1*,* 82 % identity).

#### Gustatory receptors

Six candidate GR*-*encoding transcripts were identified (Figure 5) in *I. typographus*, including putative conserved carbon dioxide receptors (GR1-3) [15]. Two GR candidates (GR1 and GR3) were identified in *D. ponderosae.* Interestingly, GR2 was not found in our *D. ponderosae* antenna-specific assembly, but was recovered from the draft genome [63] and from larval RNAseq data.

GR6 in *I. typographus* could tentatively be assigned to the trehalose receptor 1 in *T. castaneum* (67% amino acid identity)*.* Since GRs and ORs are members of the same superfamily, both were included in the same dendrogram analysis, in which GRs formed a distinct clade (Figure 5). All GRs except for ItypGR6 grouped within this clade.

#### Ionotropic receptors

**Figure 6:**
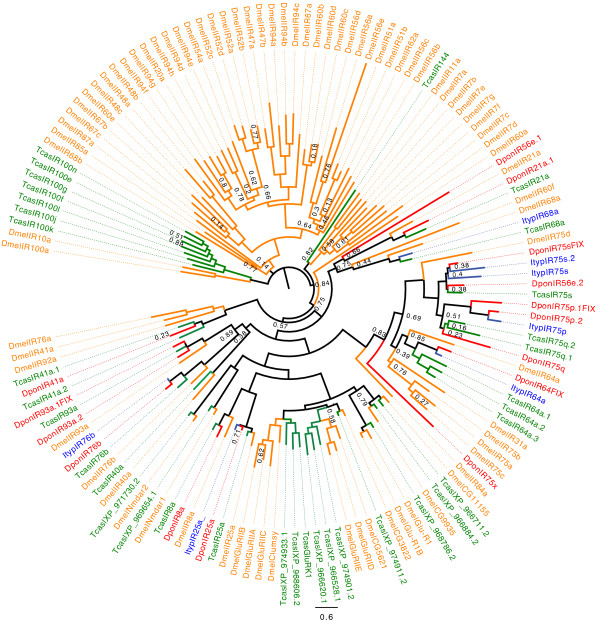
**Maximum-likelihood dendrogram based on protein sequences of candidate ionotropic receptors (IRs).** Included are IRs from *Ips typographus* (Ityp), *Dendroctonus ponderosae* (Dpon), *Tribolium castaneum* (Tcas) and *Drosophila melanogaster* (Dmel). Numbers refer to support values, which are only displayed when <; 0.9.

We identified 7 transcripts for putative ionotropic receptors in *I. typographus*, and 15 transcripts in *D. ponderosae* (Figure 6). We found bark beetle orthologues for all 10 conserved antennal IRs with representatives in *T. castaneum*[33]. However, we did not find all of them in both species. In *D. ponderosae*, we identified candidates for IR21a, IR41a, IR64a, IR76b, IR93a (a1 and a2), five members of the IR75 group, as well as the co-receptors IR25a and IR8a. Transcripts for DponIR25a, DponIR8a, DponIR75p.1FIX, DponIR75p.2, DponIR75q, and DponIR76b likely corresponded to full-length genes (or very close to), whereas all the other identified IRs were represented as partial genes. Candidate IR fragments located on 8 isotigs in *D. ponderosae* were discarded from our dendrogram analysis, as they were too short to confidently assign them unigene status. However, among these, two fragments (both 99 amino acids long) shared 72%, and 69% amino acid identity with TcasIR40a and TcasIR68a, respectively. Thus, in *D. ponderosae* it seems like orthologues for all conserved antennal IRs found in *T. castaneum*[33] were present. In contrast, we identified candidates only for IR25a, IR64a, IR68a, IR76b, and three IR75 members in *I. typographus*. Thus, several orthologues found in *D. ponderosae* and *T. castaneum* were lacking in the *I. typographus* assembly. IR8a*,* which is a broadly expressed co-receptor, necessary for odor responses [34] and present in all insects studied to date [33], was one of the receptors lacking in *I. typographus*.

## Discussion

The gene sets reported here represent significant additions to the pool of identified olfactory genes in Coleoptera. Prior to this study, members of the major chemosensory gene families in Coleoptera had been identified only from the genome of *T. castaneum* (except for the ORs also identified in the antennal transcriptome of the cerambycid, *M. caryae*). Additionally, as the genes identified here underlie the aggregation behavior that results in tree killing by mass-attack, they represent novel targets for management programs of two of the world’s most destructive forest pests.

In general, we identified somewhat larger numbers of transcripts encoding putative olfactory proteins (i.e. ORs, IRs, OBPs, and CSPs) in *D. ponderosae* than in *I. typographus*. The greater depth of the 454-sequencing and the access to Sanger data for *D. ponderosae* likely account for this difference. In addition, duplex-specific nuclease cDNA normalization (performed only for *I. typographus*) seems to result in overrepresentation of shorter full-length transcripts, which might explain the lower number of OR and IR transcripts identified in *I. typographus*, and also the absence of Orco transcripts in the transcriptome assembly. However, despite the slight difference in methodology, the GO annotation demonstrated a remarkable overall similarity in the types of genes (with respect to associated GO terms) that are expressed in the antennae of the two species. GO annotation was previously conducted for the antennal transcriptome of *Manduca sexta* moths by Grosse-Wilde et al. [27], and comparison with their data reveals a striking similarity to the bark beetles analyzed here. Indeed, several GO terms with putative relation to olfactory function showed identical (or near identical) relative abundance, suggesting a kind of across-order conservation of gene expression patterns in antennae, although the data say nothing about expression levels of the individual genes themselves.

Odorants are thought to interact with OBPs or CSPs in the sensillum lymph prior to the ligand-receptor interaction. The numbers of OBPs identified in the bark beetles (15 in *I. typographus* and 31 in *D. ponderosae*) are clearly lower than the 49 OBP-encoding genes reported in the genome of *T. castaneum*[54]*.* The same is true for the CSPs, for which we identified 6 transcripts in *I. typographus* and 11 in *D. ponderosae* compared with 20 putative CSP encoding genes in the *T. castaneum* genome [35]. However, it might be misleading to compare the number of genes identified in a genome with the number of transcripts in a specific tissue at a specific life stage. Some of the genes might, for instance, be expressed only in the larva [53,67]. Indeed, many of the identified OBPs and CSPs (ca. one third of the transcripts) in *D. ponderosae* were not identified from the antennal library, but seem to be expressed only in non-olfactory tissue. Similar patterns have been found also in other insects, suggesting that these proteins may have physiological functions independent of olfaction [41,45,68].

SNMPs are associated with pheromone-responsive OSNs in Lepidoptera and Diptera [50,51]. In *D. melanogaster*, SNMP1 was shown to be necessary for proper OSN responses to the pheromone compound *cis*-vaccenyl acetate, but not for OSN responses to food-related fruit esters [51]. Benton et al. [51] also demonstrated that SNMP was required for activation of *Heliothis virescens* (Lepidoptera) pheromone receptor HR13 by its corresponding ligand when heterologously expressed in *Drosophila* neurons. It was suggested that the hydrophobic tail of the fatty-acid derived dipteran and lepidopteran pheromone molecules necessitates the presence of SNMP. If so, that raises the question why bark beetles that do not use pheromone compounds with long hydrophobic tails [2] express SNMPs in their antennae.

The numbers of putative OR*-*encoding transcripts identified in the two bark beetles (43 in *I. typographus* and 49 in *D. ponderosae*) are close to the number reported in the antennal transcriptome of *M. caryae* (57 ORs) [29]*,* but lower than the number expressed in the head of adult *T. castaneum* (111 ORs), and much lower than the number in the *T. castaneum* genome (341 OR-encoding genes, including 79 pseudogenes) [53]*.* In other insects, the number of seemingly intact OR*-*encoding genes identified from genomes is highly variable [25], ranging from only 10 in the human body louse, *Pediculus humanus*[69], to ca. 300 in the fire ant, *Solenopsis invicta*[70]. It is not fully understood how the number of ORs relates to the ecology of an insect. In our case, one could expect that the flour beetle might have a less complex sense of smell than the forest dwelling beetles, since it has presumably adapted to an environment with a lower “semiochemical diversity” [71]. This would suggest a lower number of receptors, contrary to our results. Therefore, the chemical ecology of *T. castaneum* may be more complex than currently understood as also suggested by [53]. However, it is unknown how many of the 111 ORs that are expressed in the adult head are actually expressed in the olfactory organs of *T. castaneum*. In addition, it is likely that some bark beetle ORs have been missed in our transcriptome analysis (especially in *Ips* due to the lower sequencing depth), underestimating the true number of antennal-expressed bark beetle ORs.

Species (or taxon)-specific expansions of OR lineages are seen in most insects studied e.g. [72,73], and some of the largest expansions have been found in Hymenoptera, particularly in the jewel wasp, *Nasonia vitripennis*[74]*.* The pattern of OR lineage expansion and conservation observed in the present study likely reflects the evolutionary and ecological relatedness among the four beetle species. The beetle taxa analysed here all belong to the more derived part of Coleoptera (Cucujiformia) [75]. However, the Curculionidea (with *Ips* and *Dendroctonus*) and Tenebrionidea (with *Tribolium*) superfamilies are the two furthest separated clades within Cucujiformia, sharing a common ancestor ca. 230–240 Mya. Thus, it may come as no surprise that the ORs of these two taxa largely fall into different subgroupings in the tree. On the other hand, the Curculionidea is a sister group to the Chrysomeloidea (including the longhorns) [75] and, likewise, the closer relatedness of these taxa seems to be reflected in the OR subgroupings. Within Scolytinae, the *Ips* and *Dendroctonus* genera are separated by ca. 80 Mya [76]. However, despite the fact that *Culex* and *Aedes* mosquitoes are separated by only ca. 40 Mya [77], they show more distinct species-specific OR lineage expansions than the bark beetles [78], indicating that ecological adaptation and life cycle also play important roles in shaping the OR repertoire of a species [25]. On this note, the bark beetles and the cerambycid utilize similar types of host material, i.e. conifer trees and hardwood, respectively [2,79], whereas *T. castaneum* has been associated with human populations and stored products, for at least a few thousand years [80].

However, not all ORs were grouped in taxon-specific expansions; some subfamilies contained ORs from all four species. This might indicate preservation of ancestral functional patterns within Coleoptera, but since non-coleopteran ORs were left out from the analysis we are careful to draw any conclusions based on this finding (i.e. the clades might contain receptors also from insects outside Coleoptera).

The close clustering of OR sequences from the two bark beetles raises the question about how similar the semiochemical environment is for *I. typographus* and *D. ponderosae*. They both live in conifers and would thus be expected to share several biologically relevant compounds. Due to their status as very serious forest pests, the plant- and beetle-produced compounds that they respond to are well studied in these two species. Mainly based on a set of review papers [2,3,7,81-83], we compiled a table of all compounds that have been shown to be physiologically and/or behaviorally active in *I. typographus* and *D. ponderosae* (Additional file 5). For 29 (54%) of the 54 listed compounds, there is evidence of shared bio-activity. Not surprisingly, the host compounds show a large overlap (61%), but there is also a large overlap (56%) among pheromone compounds of beetle origin. For the non-host volatiles, the overlap is lower (40%). One might speculate that the extent of this shared “chemosphere” of semiochemicals could account for the low degree of species-specific diversifications among the bark beetle ORs and the other proteins studied here. However, functional data is required to test this hypothesis.

We identified only a small number of putative GR*-*encoding transcripts (6 in *I. typographus*; 2 in *D. ponderosae*) from the antennal transcriptomes. The identified bark beetle GRs included transcripts for carbon dioxide receptors, suggesting that the antennae of bark beetles detect carbon dioxide. In addition, the presence of GR1-3 in *I. typographus* indicates that carbon dioxide is detected by a heterotrimer receptor, like in mosquitoes, *Bombyx mori*, and *T. castaneum*[15,84]. However, GR2 was not found in the analyzed transcriptome of *D. ponderosae*. Hence, it is possible that *D. ponderosae* uses a heterodimer receptor for carbon dioxide detection (like *D. melanogaster*) [85], but it seems unlikely that expression of GR2 would have been lost in only one of the bark beetle species analyzed here.

All the conserved antennal IRs that previously were found in *T. castaneum* were also identified in *D. ponderosae*. However, some of them were missing in the *I. typographus* data. As IRs are associated with coeloconic sensilla that are relatively rare on the *Ips* antenna [86], it is possible that the missing IR transcripts are expressed only in a few neurons. A lower expression level results in a higher probability that these transcripts were missed during the random sequencing of the *Ips* cDNA, which had a lesser depth than for *D. ponderosae*. Generally in insects, the antennal IR subfamily constitutes only a portion of the total number of IRs. The others belong to the divergent IRs, a subfamily that shows species-specific expansions that are particularly large in Diptera [33]. In *D. melanogaster*, expression of divergent IRs was detected only in gustatory organs [9,33]. This is consistent with the scarcity of divergent IRs in the bark beetle antennal transcriptomes.

## Conclusions

We have carried out comprehensive analyses of the antennal transcriptomes of two major tree-killing bark beetle species. At an abstract level, the prevalence of transcript types with respect to associated GO terms was highly similar in the two beetles. In addition, we annotated members of six major gene families that encode proteins with crucial roles in chemoreception. Thus, these proteins have now been identified in three coleopteran species (four species considering the ORs). In combination with the previously published data, the gene sets identified here now allow for improved evolutionary analysis of coleopteran olfaction. We found clear expanded bark beetle-specific lineages mainly among the ORs, suggesting that in comparison to the other analyzed protein families ORs are more tightly linked to sensory specialization and adaptation to specific ecological niches and a shared space of semiochemicals.

The results from the present study will also be fundamental for future functional studies. Functional characterization is needed in order to connect the available physiological and ecological knowledge with the molecular information presented here. Identification and de-orphanization of receptor proteins in bark beetles is especially relevant, since they may represent new targets for integrated pest management strategies.

## Competing interests

The authors declare that they have no competing interests.

## Authors’ contributions

MNA and EGW contributed equally to this work, by carrying out lab work (MNA) and assemblies (EGW) for *I. typographus*, as well as bioinformatic analysis, sequence alignment, sequence editing, and data analysis for ORs, GRs, and IRs of both species. MNA drafted the manuscript. EGW constructed trees for all gene families. CIK and MMSY performed antenna-specific assembly for *D. ponderosae*, as well as bioinformatic analysis, sequence alignment, sequence editing, and data analysis for OBPs, CSPs, and SNMPs from both species. JMB performed lab work, bioinformatic analysis (*I. typographus* ORs, GRs), and data analysis. ML sequenced representative cDNA clones of *D. ponderosae* genes that appeared to be full-length for submission to NCBI. FS conducted the GO annotations and compilation of shared semiochemicals. CIK, YH, JB, BSH, and FS conceived of the study, coordinated, and designed the study. All authors contributed to study design and manuscript preparation. All authors read and approved the final manuscript

## Supplementary Material

Additional file 1 Accession numbers for genes encoding olfactory proteins that were submitted to GenBank and the Transcriptome Shotgun Assembly sequence database (TSA) at NCBI. Click here for file

Additional file 2 **Gene ontology results.** Gene ontology analyses as in Figure 1, but here represented as bar diagrams that have a higher resolution. A) Molecular function level 3 in *Ips typographus*, B) molecular function level 3 in *Dendroctonus ponderosae*, C) biological process level 2 in *I. typographus*, and D) biological process level 2 in *D. ponderosae.* Click here for file

Additional file 3 **Presence of odorant binding proteins in various tissues of *Dendroctonus ponderosae.*** Analyses of Sanger-specific data and normalized as well as non-normalized transcriptome assemblies from various body parts of *D. ponderosae* indicate that large sets of odorant binding proteins were found exclusively in non-antennal tissues. The numbers in the tables represent reads found. The clones were sequenced from both 5' and 3' directions. For further information see Keeling et al. [55]. Click here for file

Additional file 4 **Presence of chemosensory proteins in various tissues of *Dendroctonus ponderosae.*** Analyses of Sanger-specific data and normalized as well as non-normalized transcriptome assemblies from various body parts of *D. ponderosae* indicate that 4 of the 11 identified chemosensory proteins were found exclusively in non-antennal tissues. The numbers in the tables represent reads found. The clones were sequenced from both 5' and 3' directions. For further information see Keeling et al. [55]. Click here for file

Additional file 5 **Shared chemosphere of *I. typographus*and*D. ponderosae.*** List of 54 semiochemicals that are produced by the two bark beetle species, or present in their host or non-host plants and whether the compounds are active at a physiological and/or behavioral level in each species. Click here for file
