# Supplementary material for: Antennal transcriptome analysis of the chemosensory gene families in the tree killing bark beetles, Ips typographus and Dendroctonus ponderosae (Coleoptera: Curculionidae: Scolytinae)
Source: BMC Genomics. 2013 Mar 21;14:198. doi: 10.1186/1471-2164-14-198 (PMC3610139; doi:10.1186/1471-2164-14-198)

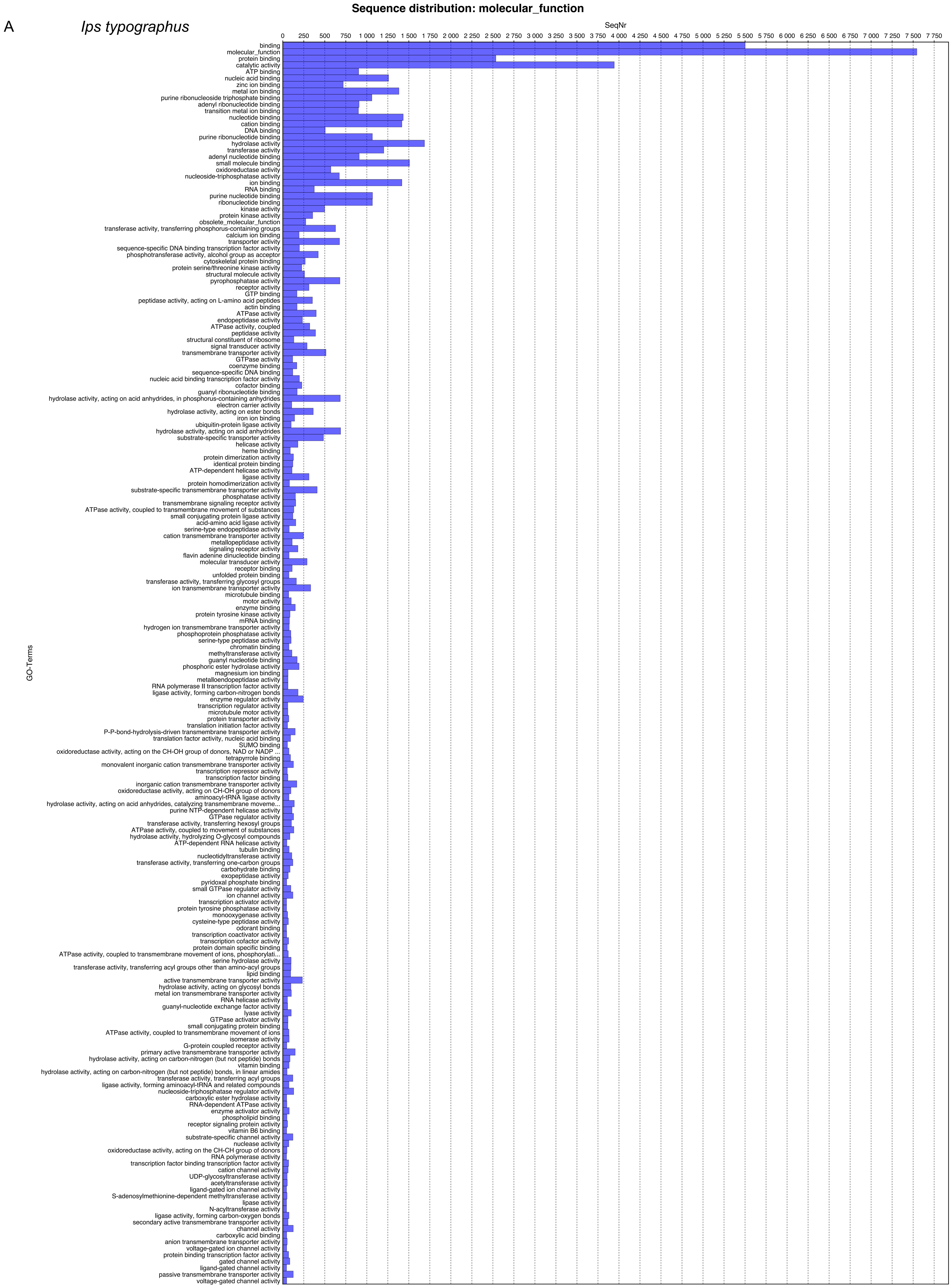

Sequence distribution: molecular_function
A
Ips typographus
SeqNr
0 250 500 750 1 000 1 250 1 500 1 750 2 000 2 250 2 500 2 750 3 000 3 250 3 500 3 750 4 000 4 250 4 500 4 750 5 000 5 250 5 500 5 750 6 000 6 250 6 500 6 750 7 000 7 250 7 500 7 750
GO-Terms
binding
molecular_function
protein binding
catalytic activity
ATP binding
nucleic acid binding
zinc ion binding
metal ion binding
purine ribonucleoside triphosphate binding
adenyl ribonucleotide binding
transition metal ion binding
nucleotide binding
cation binding
DNA binding
purine ribonucleotide binding
hydrolase activity
transferase activity
adenyl nucleotide binding
small molecule binding
oxidoreductase activity
nucleoside-triphosphatase activity
ion binding
RNA binding
purine nucleotide binding
ribonucleotide binding
kinase activity
protein kinase activity
obsolete_molecular_function
transferase activity, transferring phosphorus-containing groups
calcium ion binding
transporter activity
sequence-specific DNA binding transcription factor activity
phosphotransferase activity, alcohol group as acceptor
cytoskeletal protein binding
protein serine/threonine kinase activity
structural molecule activity
pyrophosphatase activity
receptor activity
GTP binding
peptidase activity, acting on L-amino acid peptides
actin binding
ATPase activity
endopeptidase activity
ATPase activity, coupled
peptidase activity
structural constituent of ribosome
signal transducer activity
transmembrane transporter activity
GTPase activity
coenzyme binding
sequence-specific DNA binding
nucleic acid binding transcription factor activity
cofactor binding
guanyl ribonucleotide binding
hydrolase activity, acting on acid anhydrides, in phosphorus-containing anhydrides
electron carrier activity
hydrolase activity, acting on ester bonds
iron ion binding
ubiquitin-protein ligase activity
hydrolase activity, acting on acid anhydrides
substrate-specific transporter activity
helicase activity
heme binding
protein dimerization activity
identical protein binding
ATP-dependent helicase activity
ligase activity
protein homodimerization activity
substrate-specific transmembrane transporter activity
phosphatase activity
transmembrane signaling receptor activity
ATPase activity, coupled to transmembrane movement of substances
small conjugating protein ligase activity
acid-amino acid ligase activity
serine-type endopeptidase activity
cation transmembrane transporter activity
metallopeptidase activity
signaling receptor activity
flavin adenine dinucleotide binding
molecular transducer activity
receptor binding
unfolded protein binding
transferase activity, transferring glycosyl groups
ion transmembrane transporter activity
microtubule binding
motor activity
enzyme binding
protein tyrosine kinase activity
mRNA binding
hydrogen ion transmembrane transporter activity
phosphoprotein phosphatase activity
serine-type peptidase activity
chromatin binding
methyltransferase activity
guanyl nucleotide binding
phosphoric ester hydrolase activity
magnesium ion binding
metalloendopeptidase activity
RNA polymerase II transcription factor activity
ligase activity, forming carbon-nitrogen bonds
enzyme regulator activity
transcription regulator activity
microtubule motor activity
protein transporter activity
translation initiation factor activity
P-P-bond-hydrolysis-driven transmembrane transporter activity
translation factor activity, nucleic acid binding
SUMO binding
oxidoreductase activity, acting on the CH-OH group of donors, NAD or NADP ...
tetrapyrrole binding
monovalent inorganic cation transmembrane transporter activity
transcription repressor activity
transcription factor binding
inorganic cation transmembrane transporter activity
oxidoreductase activity, acting on CH-OH group of donors
aminoacyl-tRNA ligase activity
hydrolase activity, acting on acid anhydrides, catalyzing transmembrane moveme...
purine NTP-dependent helicase activity
GTPase regulator activity
transferase activity, transferring hexosyl groups
ATPase activity, coupled to movement of substances
hydrolase activity, hydrolyzing O-glycosyl compounds
ATP-dependent RNA helicase activity
tubulin binding
nucleotidyltransferase activity
transferase activity, transferring one-carbon groups
carbohydrate binding
exopeptidase activity
pyridoxal phosphate binding
small GTPase regulator activity
ion channel activity
transcription activator activity
protein tyrosine phosphatase activity
monooxygenase activity
cysteine-type peptidase activity
odorant binding
transcription coactivator activity
transcription cofactor activity
protein domain specific binding
ATPase activity, coupled to transmembrane movement of ions, phosphorylati...
serine hydrolase activity
transferase activity, transferring acyl groups other than amino-acyl groups
lipid binding
active transmembrane transporter activity
hydrolase activity, acting on glycosyl bonds
metal ion transmembrane transporter activity
RNA helicase activity
guanyl-nucleotide exchange factor activity
lyase activity
GTPase activator activity
small conjugating protein binding
ATPase activity, coupled to transmembrane movement of ions
isomerase activity
G-protein coupled receptor activity
primary active transmembrane transporter activity
hydrolase activity, acting on carbon-nitrogen (but not peptide) bonds
vitamin binding
hydrolase activity, acting on carbon-nitrogen (but not peptide) bonds, in linear amides
transferase activity, transferring acyl groups
ligase activity, forming aminoacyl-tRNA and related compounds
nucleoside-triphosphatase regulator activity
carboxylic ester hydrolase activity
RNA-dependent ATPase activity
enzyme activator activity
phospholipid binding
receptor signaling protein activity
vitamin B6 binding
substrate-specific channel activity
nuclease activity
oxidoreductase activity, acting on the CH-CH group of donors
RNA polymerase activity
transcription factor binding transcription factor activity
cation channel activity
UDP-glycosyltransferase activity
acetyltransferase activity
ligand-gated ion channel activity
S-adenosylmethionine-dependent methyltransferase activity
lipase activity
N-acyltransferase activity
ligase activity, forming carbon-oxygen bonds
secondary active transmembrane transporter activity
channel activity
carboxylic acid binding
anion transmembrane transporter activity
voltage-gated ion channel activity
protein binding transcription factor activity
gated channel activity
ligand-gated channel activity
passive transmembrane transporter activity
voltage-gated channel activity

B *Dendroctonus ponderosae*

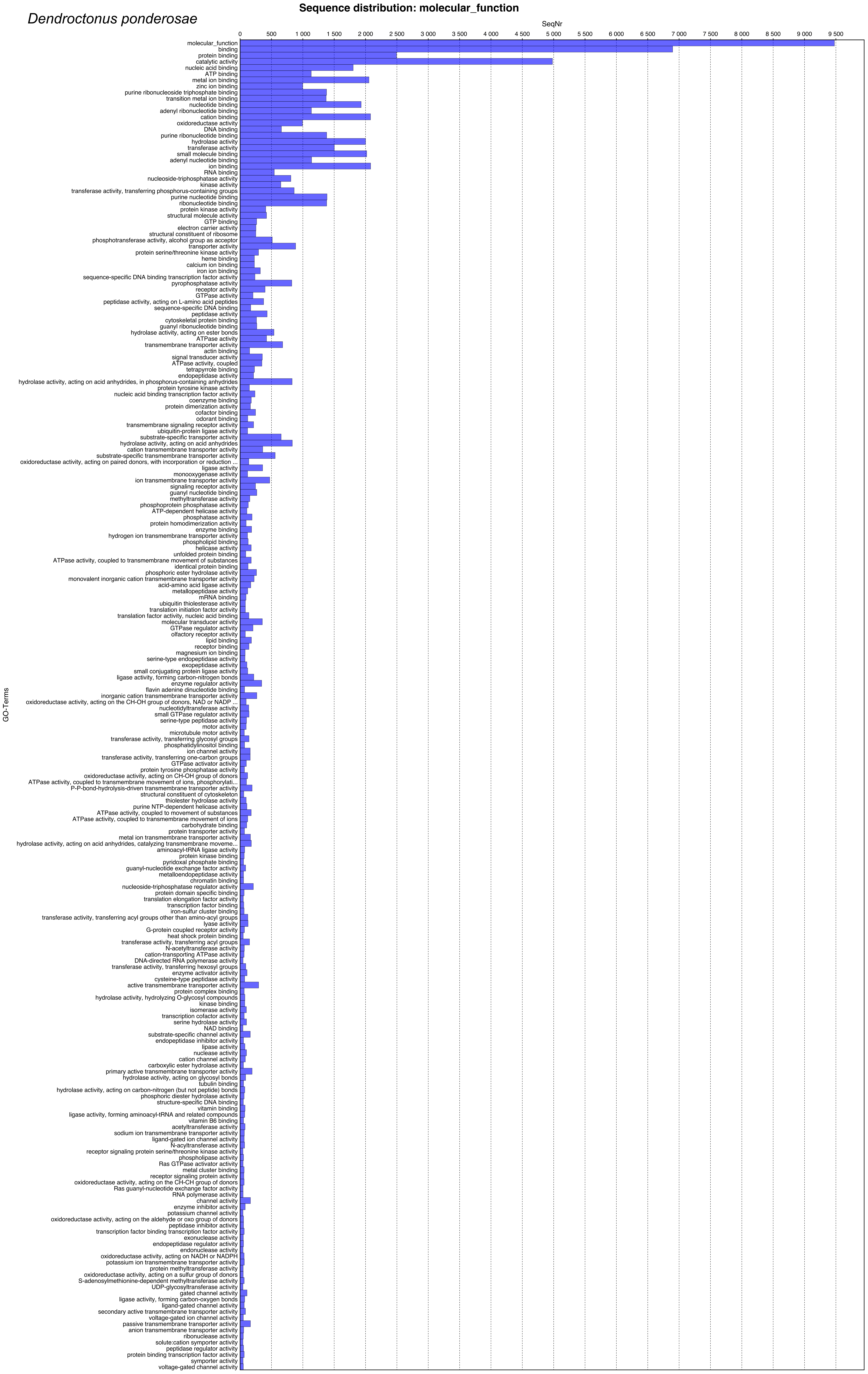

C *Ips typographus*

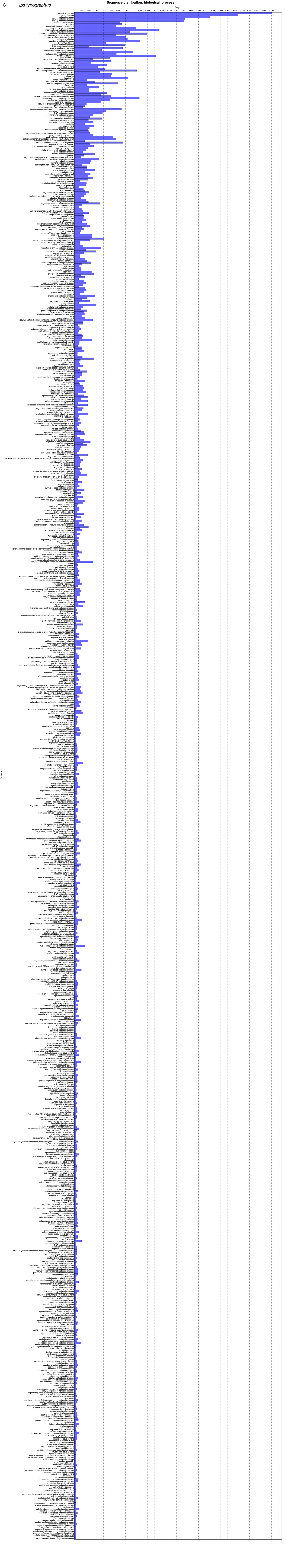

D *Dendroctonus ponderosae*

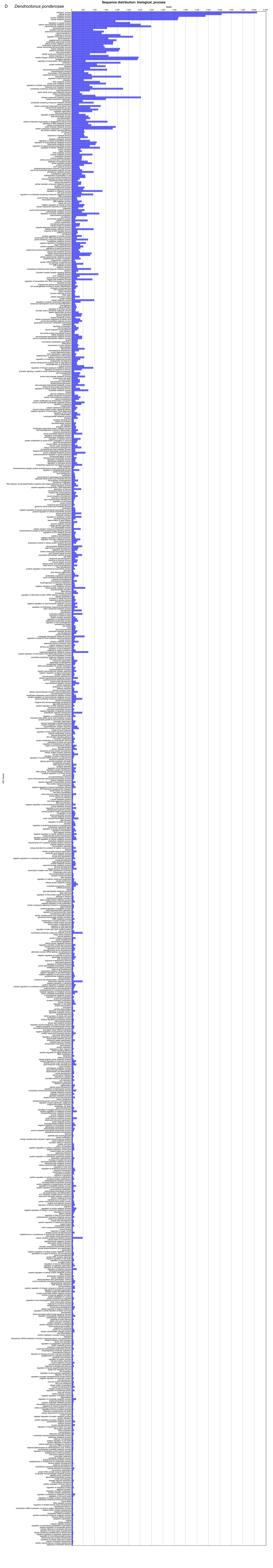

Supplement: Additional file 2 — Gene ontology results. Gene ontology analyses as in Figure 1, but here represented as bar diagrams that have a higher resolution. A) Molecular function level 3 in Ips typographus, B) molecular function level 3 in Dendroctonus ponderosae, C) biological process level 2 in I. typographus, and D) biological process level 2 in D. ponderosae. [file 1471-2164-14-198-S2.pdf]
