# Supplementary material for: Antennal transcriptome analysis of the chemosensory gene families in the tree killing bark beetles, Ips typographus and Dendroctonus ponderosae (Coleoptera: Curculionidae: Scolytinae)
Source: BMC Genomics. 2013 Mar 21;14:198. doi: 10.1186/1471-2164-14-198 (PMC3610139; doi:10.1186/1471-2164-14-198)
Supplement: Additional file 5 — Shared chemosphere of I. typographusandD. ponderosae. List of 54 semiochemicals that are produced by the two bark beetle species, or present in their host or non-host plants and whether the compounds are active at a physiological and/or behavioral level in each species. [file 1471-2164-14-198-S5.pdf]

Semiochemicals overlap

Source	Compound names*	Mountain pine beetle, *Dendroctonus ponderosae*			European Spruce Engraver, *Ips typographus*			Co-occurence
		Produced or Present[2,3,4,5]	Physiology† [2,3,4,5,6]	Behavior‡ [2,4,5,6,7,8,9]	Produced or Present	Physiology [1,6,7]	Behavior [1,5,6,7]	Strength of evidence (%)
Host	(−)-α-Pinene	+	+	+	+	+	+	100
	1,8-Cineole	?	-	?	+	+	+	0
	3-Carene	+	+	?	+	+		44
	α-Terpinene	+	+		?	-		0
	β-Phellandrene	+	+		?	-		0
	β-Pinene	+	+		+	+		44
	Camphene	+	+		+	-		22
	Camphor	+		?	+	+		22
	Estragole	?			+	+		0
	γ-terpinene	+	+		+	+		44
	Isopinocamphone	?			+	+		0
	Limonene	+	+	?	+	+		44
	Linalool	?		?	+	+	+	0
	Myrcene	+	+	+	+	+		67
	Nonanal	+	+	+	+	+		67
	para -Cymene	+	+	?	+	+		44
	Pinocamphone	+		?	+	+		22
	Pinocarvone	+		+	+	+		44
	Sabinene	+	+		+	+		44
	Styrene	?			+	+		0
	Terpinene-4-ol	?			+	+		0
	Terpinolene	+	+		+	+		44
	trans -Thujan-4-ol	?			+	+		0
Beetle								
	(+)-Ipsdienol	+	+		+	+	+	67
	(±)-Chalcogran	?			+	+	+	0
	(±)-Ipsenol	+			+	+	+	33
	2-Phenylethanol	+	+	+	+	+		67
	Acetophenone	+	+	-	+	-		22
	Amitinol	?			+	+		0
	Borneol	+	+		+?	-		0
	cis -Verbenol	+	+	+	+	+	+	100
	endo -Brevicomin	+	+	+	?	-		0
	exo-Brevicomin	+	+	+	+	+	+	100
	Frontalin	+	+	+	-	-		0
	Lanerione	+	+		-			0
	Limonene oxides (E&Z)	+	+		?	-		0
	Myrtenol	+	+		+	-		22
	trans -Verbenol	+	+	+	+	+	?	67
	Verbenone	+	+	+	+	+	+	100
Non-host (NHV)[5]								
	(*S,S*)-*trans* -Conophthorin	+	+	+	+	+	+	100
	1-Hexanol	+	+	+	+	+	+	100
	1-Octen-3-ol	+	+	+	+	+	+	100
	3-Octanol	-		-	+	+	+	0
	Benzaldehyde	+	+	+				0
	Benzyl alcohol	+	+	+				0
	E 2-Hexenal	+	+	+	?	-	-	0
	E 2-Hexenol	+	+	+	+	+	+	100
	E 3-Hexenol	?				+	+	0
	Guaiacol	+	+	+				0
	Heptanal	+	+					0
	Hexanal	+	+	+	?	-	-	0
	Salicylaldehyde	+	+	-		-		0
	Z 2-Hexenol	+	+	+	+	+	+	100
	Z 3-Hexenol	+	+	+	?	+	+	67

*) As given in publications

†) Active in single-sensillum recordings (SSR) or in gas chromatography-electroantennographic detection (GC-EAD)

‡) Active in lab and field bioassays, as attractant or anti-attractant alone or as synergist

[1 to 9]) Sources: 1) Andersson MN: Mechanisms of odor coding in coniferous bark beetles: From neuron to behavior and application. Psyche: A Journal of Entomology 2012, 2012:Article ID 149572. 2) Pureswaran DS, Borden JH: New repellent semiochemicals for three species of Dendroctonus (Coleoptera: Scolytidae). Chemoecology 2004, 14(2):67-75. 3) Pureswaran DS, Gries R, Borden JH: Antennal responses of four species of tree-killing bark beetles (Coleoptera: Scolytidae) to volatiles collected from beetles, and their host and nonhost conifers. Chemoecology 2004, 14(2):59-66. 4) Pureswaran DS, Gries R, Borden JH, Pierce Jr HD: Dynamics of pheromone production and communication in the mountain pine beetle, Dendroctonus ponderosae Hopkins, and the pine engraver, Ips pini (Say)(Coleoptera: Scolytidae). Chemoecology 2000, 10(4):153-168.
5) Schlyter F, Birgersson GA: Forest beetles. In: Pheromones of non-Lepidopteran insects associated with agricultural plants. Edited by Hardie J, Minks AK. Oxford: CAB International; 1999: 113-148. 6) Zhang Q-H, Schlyter F: Olfactory recognition and behavioural avoidance of angiosperm nonhost volatiles by conifer-inhabiting bark beetles. Agricultural and Forest Entomology 2004, 6(1):1-19. 7) Schiebe C, Jankova J, Birgersson G, Brodelius P, Schlyter F, Hansson BS: Strong antennal responses by Ips typographus L. (Scolytidae, Scolytinae) to stress related host compounds. Journal of Chemical Ecology 2013, in press. 8) Schlyter F: Odour Communication in Bark Beetles. [In Swedish]. Introductory Assay. Lund: Lund University; 1977. 9) Borden JH, Pureswaran DS, Lafontaine JP: Synergistic blends of monoterpenes for aggregation pheromones of the mountain pine beetle (Coleoptera: Curculionidae). Journal of Economic Entomology 2008, 101(4):1266-1275.

+) Existence or activity supported by positive data (empty cells= no data)

-) Negative data for existence or activity

?) Existence is likely but not proven
